# Autonomy-Supportive Teaching and Basic Psychological Need Satisfaction among School Students: The Role of Mindfulness

**DOI:** 10.3390/ijerph16142599

**Published:** 2019-07-21

**Authors:** Chunxiao Li, Ying Hwa Kee, Leng Chee Kong, Liye Zou, Ka Lok Ng, Hong Li

**Affiliations:** 1 National Institute of Education, Nanyang Technological University, Singapore 637616, Singapore; 2 Department of Health and Physical Education, The Education University of Hong Kong, Hong Kong, China; 3 Lifestyle (Mind-Body Movement) Research Center, School of Psychology, Shenzhen University, Shenzhen 518060, China; 4 Shenzhen Key Laboratory of Affective and Social Cognitive Science, School of Psychology, Shenzhen University, Shenzhen 518060, China; 5 Shenzhen Institute of Neuroscience, Shenzhen University, Shenzhen 518060, China; 6 Institute for Brain and Psychological Sciences, Sichuan Normal University, Chengdu 610066, China

**Keywords:** autonomy support, present moment, basic psychological needs, physical education, school

## Abstract

Grounded in self-determination theory, the purpose of this study was to investigate the relationships between autonomy-supportive teaching, mindfulness, and basic psychological need satisfaction/frustration. Secondary school students (*n* = 390, *M*age = 15) responded to a survey form measuring psychological constructs pertaining to the research purpose. A series of multiple regression analysis showed that autonomy-supportive teaching and mindfulness positively predicted need satisfaction and negatively predicted need frustration. In addition, the associations between autonomy-supportive teaching and need satisfaction/frustration were moderated by mindfulness. Students higher in mindfulness were more likely to feel need satisfaction and less likely to experience need frustration, even in a low autonomy-supportive teaching environment. These results speak to the relevance of creating autonomy-supportive teaching environments and highlight mindfulness as a potential pathway to basic psychological need satisfaction in educational settings.

## 1. Introduction

A physical education (PE) class is a venue where a wide range of learning outcomes such as developing an active lifestyle, acquiring motor skills, and cultivating positive values are attained. Importantly, optimal teaching approaches such as autonomy-supportive behaviors (e.g., taking students’ perspectives, offering choices to students, and providing rationales to decision making) play a significant role in promoting learning outcomes [1,2]. However, instead of using autonomy-supportive behaviors, some teachers may choose to use controlling teaching behaviors (e.g., asking students to perform a task in a fixed way, punishing students for making mistakes, and failing to provide rationales for requested actions), which are found to result in undesirable learning outcomes [3]. Interestingly, the same autonomy-supportive or controlling teaching behavior may have a different impact on different students [4]. For example, some students become despondent and ruminative when they are punished by teachers, while others may accept the accompanying negative emotions of punishment readily. The different reactions bespeak of intra-psychic factors at play beyond teaching behaviors in PE contexts.

Recently, one intrapersonal factor that has received increasing attention is mindfulness. Mindfulness refers to “a receptive attention to and awareness of present events and experience” [5]. Distinguishing from other personality traits (e.g., openness, grit, and resilience), mindfulness has been researched as a dispositional characteristic. Mindfulness can also be cultivated through intended mindfulness practices such as those found in mindfulness-based training programs [6]. Mounting evidence has indicated that mindfulness contributes to physical and psychological benefits across both clinical and nonclinical populations (e.g., [7,8]). As such, some schools are training their teachers and students in mindfulness [9]. However, there is little evidence regarding the role of dispositional mindfulness in the relationship between autonomy-supportive teaching and its outcomes. Grounded in self-determination theory (SDT) [10], this research aims to examine the moderating effect of mindfulness on autonomy-supportive teaching and basic psychological needs satisfaction within the PE context.

### 1.1. Basic Psychological Needs Theory

Basic psychological needs theory (BPNT) [10], a sub-theory under SDT, posits that autonomy, competence and relatedness are universal psychological needs of human beings and when satisfied will conduce toward well-being; and when frustrated will lead to ill-being. Autonomy satisfaction is experienced when the individual feels a sense of choice and volition when carrying out an activity. In contrast, autonomy frustration occurs when the individual feels controlled through internal or external pressures [11]. Competence satisfaction occurs when the individual feels effective and capable of achieving desired outcomes. When competence is frustrated, the individual feels a sense of failure and has doubt about one’s ability [11]. Relatedness is satisfied when the individual feels a sense of connectedness with others. When relatedness is frustrated, the individual feels a sense of isolation and loneliness [11].

In line with the postulations of BPNT [10], research has shown that basic psychological need satisfaction enhances and need frustration hampers wellness and full functioning. For example, the study of Gunnell et al. [12] in the physical activity context found changes in need satisfaction to positively predict positive affect and subjective vitality, and negatively predict negative affect. Additional analyses also revealed that changes in need frustration predicted negative affect above the contributions made by need satisfaction. Similarly, among students in PE classes, their perceptions on need satisfaction positively predicted positive affect, knowledge in sport, sports performance and intention to continue with sports participation. In contrast, need frustration corresponded negatively with positive affect and knowledge in sport [13].

### 1.2. Autonomy-Supportive Teaching and Needs Satisfaction

Given the importance of the basic psychological needs of autonomy, competence and relatedness in the promotion of wellness and positive functioning, it is therefore a worthwhile endeavor to understand how basic psychological needs are fostered and frustrated. SDT specifies the social conditions (e.g., teachers’ interpersonal behaviors or teaching behaviors) that can foster or frustrate basic psychological needs satisfaction [10]. Brown and Ryan [14] further proposed that the basic psychological needs can be facilitated both through autonomy-supportive teaching (without factor) and through mindfulness (within factor).

In the PE domain, there are numerous empirical supports for the predictive value of autonomy-supportive teaching on basic psychological needs satisfaction (i.e., the without factor). For example, Shen et al. [15] reported that perceived autonomy-support significantly predicted students’ need satisfaction. Similarly, Behzadnia et al. [13] found that perceived autonomy-supportive teaching positively correlated with need satisfaction and was inversely associated with need frustration among students. While there is a plethora of research studies on the efficacy of autonomy-support teaching on basic psychological needs satisfaction, less is known about the within factor―mindfulness―on it. Even more scant is the literature on the interactive effect of both the without and within factors―autonomy-supportive teaching and mindfulness—on the satisfaction of basic psychological needs. We shall now turn our attention to how basic psychological needs could be nurtured from within, through mindfulness.

### 1.3. Mindfulness, Autonomy-Supportive Teaching and Needs Satisfaction

Brown and Ryan [14] theorized that just as social conditions can foster or frustrate basic psychological needs satisfaction, so too can mindfulness at the personal level (i.e., receptive attention and awareness of present moment). Attention and awareness may hinder basic psychological needs satisfaction through two pathways: (a) cognitively, due to attentional limits, and (b) motivationally, due to motivational selectivity [14]. Cognitively, we are normally consciously aware of only a small aspect of our perceptions and actions [16], and much of our perceptions and actions occur automatically without conscious awareness (e.g., [17,18]). Motivationally, we tend to place high priority on information that is relevant to self-concept preservation and to give low priority to accurate self-knowledge [19]. The attentional limits and motivational selectivity biases may hinder the receptivity to events and experiences (e.g., teachers’ positive teaching behaviors) that allow for fulfilling the satisfaction of one’s psychological needs.

Researchers (e.g., [14,20,21]) suggest that mindfulness may enhance one’s receptivity to events and experiences. When there is mindful nonjudgmental awareness of one’s inherent needs, ego-involvement is less likely to dominate intra-psychic interactions; as a result, one is more likely to act autonomously. In addition, when mindful, the individual has greater capacity to perceive feedback as being informational rather than controlling, because the individual is less likely to feel ego-involved in the feedback. Thus, mindfulness allows for feedback to enhance autonomy and competence [20,21]. On the satisfaction of relatedness, Rigby et al. [20] explained that when one is able to be fully in the moment, unburdened by rigidity in thinking, defensive ego protections or other preconceptions that people often bring to interpersonal interaction, one would be in a position to respond connectedly. In this manner, mindfulness may facilitate the experience of relatedness.

In sum, even when the external social environments provide an optimal motivational climate (e.g., autonomy-supportive teaching), to harness the motivational advantage, one still needs to be internally aware of one’s needs and reflectively consider one’s behavior to fit in with one’s needs [14]. In this way, mindfulness, a quality of receptive attention to and awareness of present events and experience can conduce towards informational feedback from autonomy-supportive behaviors. In other words, mindfulness is related to greater need satisfaction and lower need frustration. Accumulating research has provided some empirical support for the direct relationship between mindfulness and needs satisfaction/frustration across university students, preservice teachers, and employees (e.g., [21,22,23]). Moreover, there is initial evidence showing that mindfulness moderated the negative relationship between managers’ autonomy support and need frustration among employees. That is, mindfulness was found to buffer the negative relationship between autonomy support and need frustration. However, the interactive effect between mindfulness and autonomy support on need satisfaction was not significant [21], suggesting a need to further examine this interactive effect and probably test it in a different context as an effort to generalize research findings.

### 1.4. Aims and Hypotheses

To the best of our knowledge, there is currently no published study on the interactive effect between mindfulness and autonomy-supportive teaching on basic psychological needs satisfaction. Examining this possible interactive effect in the classroom setting is important for understanding why the same teaching behavior may have a different impact on different students. This is also relevant to the reason of using mindfulness interventions in schools [9]. The finding may provide a new perspective on why it is necessary to train students in mindfulness. The aim of this research was therefore to investigate the relationships between autonomy-supportive teaching, need satisfaction/frustration, and mindfulness in secondary school students. Based on the above literature review and reasoning, it was hypothesized that autonomy-supportive teaching and mindfulness would be positively associated with need satisfaction (Hypothesis 1a) and negatively associated with need frustration (Hypothesis 1b). Furthermore, it was expected that the positive association between autonomy-supportive teaching and need satisfaction would be stronger when mindfulness is higher (Hypothesis 2a). In addition, the negative relationship between autonomy-supportive teaching and need frustration would be weaker when participants have a higher mindfulness level (Hypothesis 2b).

## 2. Materials and Methods

### 2.1. Participants

A total of 390 Chinese secondary school students from Hong Kong participated in this survey research. Participants were recruited from 17 classes (Grade 7–12) of three public secondary schools. Participants had a mean age of 15 years (SD = 2.05) and there were more female participants than males (boy = 164, girl = 226). All of them were able to read and write Chinese language.

### 2.2. Measures

#### 2.2.1. Autonomy-Supportive Teaching

The Chinese version of the Need-Supportive Teaching Style Scale for PE [24] was used to assess participants’ perceived autonomy-supportive teaching behaviors from their PE teachers. The scale consists of ten items and measures three types of need-supportive teaching: involvement (3 items; e.g., “My teacher spends time with me”), autonomy (3 items; e.g., “My teacher gives me a lot of choices”), and structure (4 items; e.g., “My teacher makes sure I understand before he/she goes on”). Participants provided responses on a 7-point Likert scale (1 = “strongly disagree”, 7 = “strongly agree”). A mean scale score was computed for subsequent analyses and a higher score suggests a greater level of autonomy-supportive teaching. The scale and its subscales demonstrated good internal reliability with the present sample (α = 0.82 to 0.95).

#### 2.2.2. Need Satisfaction

The Chinese version of the Psychological Needs Satisfaction Scale in PE [25] employed to measure participants’ perceived need satisfaction in PE classes. This 10-item scale measures three types of basic psychological need satisfaction: autonomy (4 items; e.g., “I have opportunities to express my views and thoughts in my PE classes”), competence (3 items; e.g., “I have the ability to perform well in my PE classes”), and relatedness (3 items; e.g., “I get along well with the people in my PE classes”). Participants provided responses on a 7-point Likert scale (1 = “strongly disagree”, 7 = “strongly agree”). A mean scale score was used for further analyses and a higher score indicates a greater level of need satisfaction. The scale and subscales showed good internal reliability in the current study (α = 0.81 to 0.93).

#### 2.2.3. Need Frustration

The Chinese version of the Psychological Needs Thwarting Scale in PE [26] was applied to measure participants’ perceived need frustration in PE classes. The scale consists of three 3-item subscales: autonomy need frustration (e.g., “I feel pushed to behave in certain ways in my PE classes”), competence need frustration (e.g., “There are situations in which I am made to feel inadequate in my PE classes”), and relatedness need frustration (e.g., “I feel some people in my PE classes do not like me much”). Participants provided responses on a 7-point Likert scale (1 = “strongly disagree”, 7 = “strongly agree”). A mean scale score was calculated for subsequent analyses and a higher score represents a greater level of need frustration. The scale and subscales demonstrated good internal reliability in the current study (α = 0.83 to 0.94).

#### 2.2.4. Mindfulness

The Chinese version of the Mindfulness Attention Awareness Scale [27] was used to assess participants’ trait mindfulness. The scale has 15 items and a sample item is “I tend not to notice feelings of physical tension or discomfort until they really grab my attention.” Participants rated the scale items on a 6-point Likert scale (1 = “almost always”, 6 = “almost never”). The mean scale score was computed for further analyses and a higher score reflects a greater level of trait mindfulness. The scale demonstrated good internal reliability with the present sample (α = 0.90).

### 2.3. Procedure

Ethical approval was granted by the Human Research Ethics Committee of The Education University of Hong Kong. School principals of the three secondary schools were contacted via email to obtain permission to conduct this study as well as to invite their students to participate in this research. Upon obtaining permission from school principals, written informed consent was obtained from students and their parents prior to data collection. The survey forms were administrated to students after their PE classes by research assistants. The research assistants emphasized the voluntary and confidentiality of participation in this research. Of 417 students from 17 classes invited, 390 provided responses (response rate = 93.5%). Participants spent approximately 10 min to complete the survey.

### 2.4. Data Analysis

Descriptive statistics (*M*s, SDs, and intercorrelations) for the major study variables were computed. As the participants were nested within 17 classes, intra-class correlations (ICCs) for outcome variables were calculated to decide whether multi-level analysis should be considered. Given that ICC values were smaller than 0.10 (need satisfaction/frustration = 0.09/0.08), “class-level” effects were not considered in subsequent analyses [28]. Two multiple regressions were conducted to test: (a) Whether autonomy-supportive teaching and mindfulness can predict need satisfaction (Hypothesis 1a)/frustration (Hypothesis 1b); and (b) whether mindfulness can moderate autonomy-supportive teaching in predicting need satisfaction (Hypothesis 2a) and need frustration (Hypothesis 2b). For both regressions, covariates (age and gender) were entered in the first step, autonomy-supportive teaching and mindfulness were entered in the second step, and the two-way interaction term (autonomy-supportive teaching × mindfulness) was entered in the final step. All the analyses were conducted in IBM SPSS Statistics 25 (IBM, Armonk, NY, USA).

## 3. Results

Internal reliability and descriptive statistics for the major study measures are presented in Table 1. Gender and age were not significantly associated with autonomy-supportive teaching, mindfulness, need satisfaction, and need frustration except for a significant association between age and need frustration (*r* = −0.12, *p* = 0.02). Autonomy-supportive teaching was significantly related to mindfulness (*r* = 0.28, *p* < 0.001), need satisfaction (*r* = 0.60, *p* < 0.001), and need frustration (*r* = −0.38, *p* < 0.001). In addition, mindfulness was significantly associated with need satisfaction (*r* = 0.35, *p* < 0.001) and need frustration (*r* = −0.53, *p* < 0.001).

Table 2 presents the results of multiple regression analysis. In step 1, gender and age did not significantly predict need satisfaction. Autonomy-supportive teaching (β = 0.55, *p* < 0.001) and mindfulness (β = 0.19, *p* < 0.001) were significant predictors of need satisfaction in step 2. In step 3, the interaction of autonomy-supportive teaching and mindfulness significantly predicted need satisfaction (β = 0.08, *p* = 0.048). In addition, autonomy-supportive teaching (β = 0.54, *p* < 0.001) and mindfulness (β = 0.21, *p* < 0.001) remained as significant predictors. Thus, Hypothesis 1a is confirmed.

**Figure 1:**
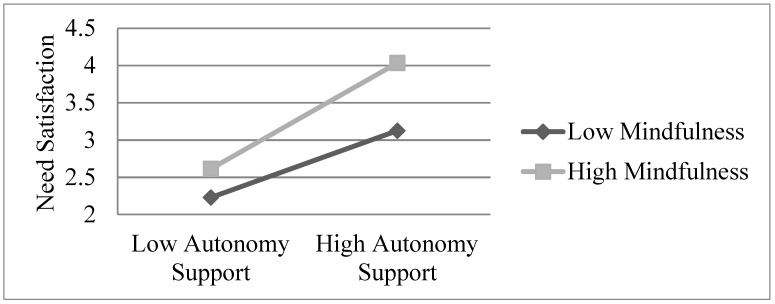
Moderation effect of mindfulness on the relationship between autonomy support and need satisfaction.

All the predictors explained 41% of the total variance in need satisfaction. Given the significant interaction effect found, a simple slope analysis was conducted and the interaction effect was plotted [29]. It was found that the positive relationship between autonomy-supportive teaching and need satisfaction was positive at both low (*M* − 1 *SD*; B = 0.49, *p* < 0.001) and high levels (*M* + 1 *SD*; B = 0.67, *p* < 0.001) of mindfulness. These findings support Hypothesis 2a in that the positive association between autonomy-supportive teaching and need satisfaction was strengthened by the presence of high mindfulness (see Figure 1).

Age (β = −0.12, *p* = 0.02) but not gender significantly predicted need frustration in step 1 (see Table 2). In step 2, autonomy-supportive teaching (β = −0.26, *p* < 0.001) and mindfulness (β = −0.45, *p* < 0.001) were found to significantly predict need frustration. Finally, autonomy-supportive teaching (β = −0.27, *p* < 0.001) and mindfulness (β = −0.43, *p* < 0.001) were significant predictors of need frustration in step 3. Moreover, the interaction of autonomy-supportive teaching and mindfulness was a significant predictor of need frustration (β = 0.12, *p* = 0.02). Thus, Hypothesis 1b is supported.

**Figure 2:**
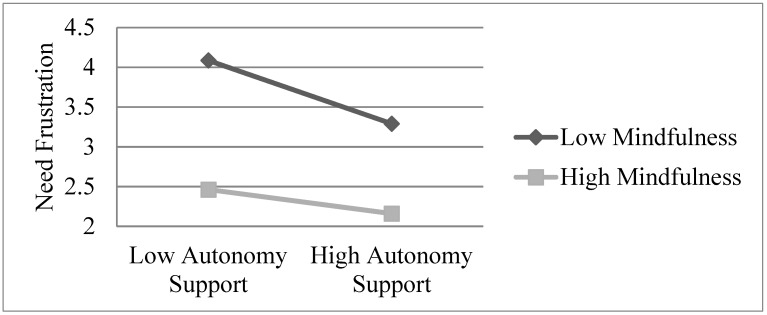
Moderation effect of mindfulness on the relationship between autonomy support and need frustration.

All the predictors explained 36% of the total variance in need frustration. Following the significant interaction effect, the result of simple slope analysis revealed that the relationship between autonomy-supportive and need frustration was negative at both low (*M* − 1 *SD*; B = −0.36, *p* < 0.001) and high levels (*M* + 1 *SD*; B = −0.19, *p* < 0.001) of mindfulness. These findings are in line with Hypothesis 2b, in which the negative relationship between autonomy-supportive teaching and need frustration was attenuated by the presence of high mindfulness (see Figure 2).

**Figure 3:**
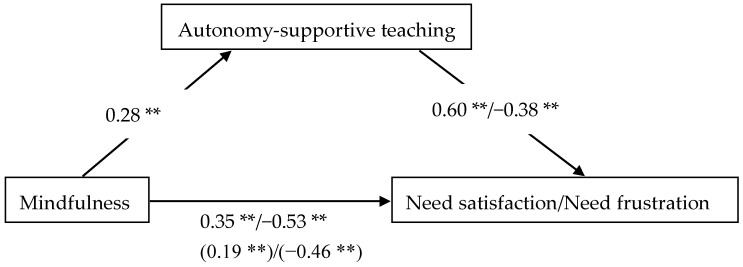
Standardized regression coefficients for the direct relationship between mindfulness and need satisfaction/frustration as mediated by autonomy-supportive teaching. The indirect standardized regression coefficients are represented in parentheses. ** *p* < 0.01.

Additional analyses were conducted to explore whether autonomy-supportive teaching can mediate the relationship between mindfulness and need satisfaction/frustration. Mediation analyses with bootstrapping (5000 samples) were used to generate bias corrected confidence intervals (CIs) [30]. The indirect effect of mindfulness on need satisfaction was significant, β = 0.15, 95%CI [0.10, 0.21], SE = 0.05. In addition, the indirect effect of mindfulness on need frustration was also significant, β = −0.07, 95%CI [−0.12, −0.03], SE = 0.02. These findings together with those results presented in Table 2 suggested that autonomy-supportive teaching was a partial mediator in the relationship between mindfulness and need satisfaction/frustration (see Figure 3).

## 4. Discussion

Drawing on self-determination theory [10], we investigated the relationships among autonomy-supportive teaching, need satisfaction/frustration and mindfulness. Supporting the proposed hypotheses, we found that autonomy-supportive teaching and mindfulness were positively related to need satisfaction (Hypothesis 1a), whereas autonomy-supportive teaching and mindfulness negatively predicted need frustration (Hypothesis 1b). More significantly, mindfulness moderated the relationship between autonomy-supportive teaching and need satisfaction/frustration (Hypotheses 2a and 2b).

In line with BPNT [10], we found that autonomy-supportive teaching positively predicted need satisfaction and was inversely related to need frustration. These findings are in accordance with early research (e.g., [1,15,31]) and highlight the satisfaction of basic psychological needs depends on the quality of autonomy-supportive teaching. In the present study, we found that autonomy-supportive teaching better predicted need satisfaction (β = 0.55) than need frustration (β = −0.26). This could be because there are two parallel pathways or processes from teaching climates to the three basic psychological needs [11]. Namely, a “bright” pathway (e.g., from autonomy-supportive teaching to need satisfaction) and a “dark” process (e.g., from controlling teaching to need frustration). These two pathways have differential antecedents of basic psychological needs satisfaction, with autonomy-supportive behaviors mainly benefitting need satisfaction while controlling behaviors would be more strongly related to need frustration [32].

Consistent with our hypotheses, mindfulness was found to positively predict need satisfaction and negatively predict need frustration. These results are in line with earlier research conducted in other contexts, including tertiary education and organization [21,22,23]. When mindful, a student has an enhanced awareness of the window of opportunity to choose the specifics of action. In so doing, one would be more likely to act in a way that is congruent to his/her own choice (autonomy), consider feedback informational (competence), and attend to interpersonal interactions (relatedness). Thus, mindfulness as a dispositional factor can facilitate or frustrate need satisfaction at the personal level.

Our study is unique in showing the moderation role of mindfulness on need satisfaction/frustration. Specifically, we found that the positive association between autonomy-supportive teaching and need satisfaction was stronger when mindfulness was higher. Our result suggests that students higher in mindfulness, in comparison to those with lower mindfulness, reported a stronger perception of autonomy-supportive teaching climates and need satisfaction. There is a likelihood that they were more aware of their teachers’ autonomy-supportive efforts and thus benefited in terms of motivational advantages. However, in a previous study on employees by Schultz et al. [21], contrary to our finding, mindfulness did not moderate managerial autonomy-support on need satisfaction at work. The inconsistent findings highlight the value of conducting the present research. In addition, it is unclear what contributes to the inconsistency, suggesting the need to explore possible reasons in future research.

On the other hand, our result is consistent with Schultz et al. [21] research in that both studies showed that mindfulness played a protective role in mitigating the effects of non-supportive contexts on need frustration. The openness and awareness characterized by mindfulness are believed to enhance healthy emotional regulation, decrease maladaptive coping patterns (e.g., rumination), and reduce critical judgement. Subsequently, these mindfulness-related qualities would translate to reduced experience of need frustration [21]. For example, a mindful student is more likely to view non-supportive teaching behaviors (e.g., lack of skill feedback) in a nonjudgmental manner, and that may reduce the negative impact on his/her sense of skill competency and connectedness with the teacher. Thus, mindfulness seems to be critical in attenuating need frustration.

### 4.1. Practical Implications

The present study findings affirm the necessity of creating autonomy-supportive teaching environments (e.g., listening to students’ opinions, offering choices to students, and acknowledging student feelings) to facilitate students’ need satisfaction in the PE classrooms [24,31]. In addition to training PE teachers to adopt a positive teaching climate (e.g., [2,31]), our findings provide a new perspective in enhancing students’ need satisfaction. That is, since mindfulness practices are useful in cultivating students’ mindfulness skills, such implementation may increase the receptivity to PE teachers’ positive teaching behaviors as well as decrease negative responses to non-supportive or controlling teaching climates. Nowadays, a number of school-based mindfulness programs (e.g., mindfulness-based yoga interventions, mindfulness eating, and guided meditation) are available for the training purpose [9].

### 4.2. Limitations and Future Research Directions

Despite the unique empirical contributions and practical implications, the present study has several limitations that must be highlighted. First, as the participants were conveniently sampled from three secondary schools in Hong Kong, our results may not be generalized into all secondary school students and other populations. Future studies are warranted to replicate the study findings with a more presentative sample and other groups such as primary school students. Second, we employed a cross-sectional survey design, which does not permit us to infer causal relations. A longitudinal or experimental study should be used to clarify directional effects in future. Third, we fully relied on using self-reports to measure the interested constructs, resulting in common method effects (i.e., flattened correlation coefficients). Using of alterative measures (e.g., an observational tool of autonomy-supportive teaching) can address this limitation. Third, a recent study found that coaches’ self-reported autonomy-supportive behaviors were not aligned with athletes’ perceptions [33]. Similarly, there might be disagreements between teachers’ reported autonomy-supportive teaching and students’ perceptions. It would be also interesting to examine whether teacher mindfulness can attenuate the potential disagreements [34]. Finally, as there are both “bright” and “dark” pathways from interpersonal styles to the three basic psychological needs [32], future research may test the interactive effect of controlling teaching and mindfulness on need satisfaction/frustration. In addition, additional outcomes such as student engagement and satisfaction as well as other health outcomes may also be included.

## 5. Conclusions

Our study provided the initial evidence on the interactive effect between mindfulness and autonomy-supportive teaching on basic psychological needs satisfaction. The study findings show that the same PE teaching climate can be translated to different experience of basic psychological need satisfaction due to varying mindfulness levels among students. Our findings speak to the relevance of creating autonomy-supportive climates and highlight mindfulness as a potential pathway to basic psychological need satisfaction in educational settings. Providing autonomy-supportive teaching alone may not be as effective in facilitating students’ need satisfaction without cultivating their mindfulness.

## Figures and Tables

**Table 1:** Internal reliability and descriptive statistics for the study variables.

Measures	1.	2.	3.	4.	5.	6.
1. Age	—					
2. Gender	−0.09	—				
3. Autonomy-supportive teaching	−0.02	−0.03	—			
4. Mindfulness	0.02	0.03	0.28 **	—		
5. Need satisfaction	0.08	−0.04	0.60 **	0.35 **	—	
6. Need frustration	−0.12 *	−0.03	−0.38 **	−0.53 **	−0.50 **	—
*M*	15.00	—	5.28	4.62	4.70	2.41
*SD*	2.05	—	1.07	0.72	1.14	1.12
α	—	—	0.95	0.90	0.93	0.94

Note. ** *p* < 0.01, * *p* < 0.05.

**Table 2:** Results of multiple regression analyses.

Step	Predictor	Need satisfaction				Need frustration			
		B	β	*R* ^2^	Δ*R*^2^	B	β	*R* ^2^	Δ*R*^2^
1	(Constant)	4.13 **				3.46 **			
	Gender	−0.07	−0.03	0.01		−0.08	−0.04	0.02 *	
	Age	0.04	0.07			−0.07 *	−0.12 *		
2	(Constant)	−0.46				8.08 **			
	Gender	−0.05	−0.02	0.40 **	0.39 **	−0.06	−0.03	0.35 **	0.34 **
	Age	0.05 *	0.08 *			−0.06 **	−0.12 **		
	Autonomy-supportive teaching	0.59 **	0.55 **			−0.27 **	−0.26 **		
	Mindfulness	0.31 **	0.19 **			−0.71 **	−0.45 **		
3	(Constant)	−0.56				7.96 **			
	Gender	−0.05	−0.02	0.41 *	0.01 *	−0.06	−0.03	0.36 *	0.01 *
	Age	0.04	0.08			−0.07 **	−0.12 **		
	Autonomy-supportive teaching	0.58 **	0.54 **			−0.28 **	−0.27 **		
	Mindfulness	0.34 **	0.21 **			−0.67 **	−0.43 **		
	Autonomy-supportive teaching × Mindfulness	0.10 *	0.08 *			0.12 *	0.10 *		

Note. ** *p* < 0.01, * *p* < 0.05.
